# Post‐mastectomy radiotherapy for women with early breast cancer and one to three positive lymph nodes

**DOI:** 10.1002/14651858.CD014463.pub2

**Published:** 2023-06-16

**Authors:** Rashmi Verma, Mihir Chandarana, Jessica Barrett, Carmel Anandadas, Sreekumar Sundara Rajan

**Affiliations:** Breast SurgeryLancashire Teaching Hospitals NHS Foundation TrustChorleyUK; Breast SurgeryHEE East MidlandsLeicesterUK; MRC Biostatistics UnitUniversity of CambridgeCambridgeUK; Clinical OncologyChristie NHS Foundation TrustManchesterUK; Breast SurgeryUniversity Hospitals Birmingham NHS Foundation TrustBirminghamUK

**Keywords:** Female, Humans, Breast Neoplasms, Breast Neoplasms/drug therapy, Breast Neoplasms/radiotherapy, Breast Neoplasms/surgery, Combined Modality Therapy, Lymph Nodes, Lymph Nodes/pathology, Mastectomy, Neoplasm Recurrence, Local

## Abstract

**Background:**

Continual improvement in adjuvant therapies has resulted in a better prognosis for women diagnosed with breast cancer. A surrogate marker used to detect the spread of disease after treatment of breast cancer is local and regional recurrence. The risk of local and regional recurrence after mastectomy increases with the number of axillary lymph nodes affected by cancer. There is a consensus to use radiotherapy as an adjuvant treatment after mastectomy (postmastectomy radiotherapy (PMRT)) in women diagnosed with breast cancer and found to have disease in four or more positive axillary lymph nodes. Despite data showing almost double the risk of local and regional recurrence in women treated with mastectomy and found to have one to three positive lymph nodes, there is a lack of international consensus on the use of PMRT in this group.

**Objectives:**

To assess the effects of PMRT in women diagnosed with early breast cancer and found to have one to three positive axillary lymph nodes.

**Search methods:**

We searched the Cochrane Breast Cancer Group's Specialised Register, CENTRAL, MEDLINE, Embase, the World Health Organization (WHO) International Clinical Trials Registry Platform (ICTRP) and ClinicalTrials.gov up to 24 September 2021.

**Selection criteria:**

We included randomised controlled trials (RCTs). The inclusion criteria included women diagnosed with breast cancer treated with simple or modified radical mastectomy and axillary surgery (sentinel lymph node biopsy (SLNB) alone or those undergoing axillary lymph node clearance with or without prior SLNB). We included only women receiving PMRT using X‐rays (electron and photon radiation), and we defined the radiotherapy dose to reflect what is currently being recommended (i.e. 40 Gray (Gy) to 50 Gy in 15 to 25/28 fractions in 3 to 5 weeks. The included studies did not administer any boost to the tumour bed. In this review, we excluded studies using neoadjuvant chemotherapy as a supportive treatment before surgery.

**Data collection and analysis:**

We used Covidence to screen records. We collected data on tumour characteristics, adjuvant treatments and the outcomes of local and regional recurrence, overall survival, disease‐free survival, time to progression, short‐ and long‐term adverse events and quality of life. We reported on time‐to‐event outcome measures using the hazard ratio (HR) and subdistribution HR. We used Cochrane's risk of bias tool (RoB 1), and we presented overall certainty of the evidence using the GRADE approach.

**Main results:**

The RCTs included in this review were subgroup analyses of original RCTs conducted in the 1980s to assess the effectiveness of PMRT. Hence, the type and duration of adjuvant systemic treatments used in the studies included in this review were suboptimal compared to the current standard of care.

The review involved three RCTs with a total of 829 women diagnosed with breast cancer and low‐volume axillary disease. Amongst the included studies, only a single study pertained to the modern‐day radiotherapy practice. The results from this one study showed a reduction of local and regional recurrence (HR 0.20, 95% confidence interval (CI) 0.13 to 0.33, 1 study, 522 women; low‐certainty evidence) and improvement in overall survival with PMRT (HR 0.76, 95% CI 0.60 to 0.97, 1 study, 522 women; moderate‐certainty evidence). One of the other studies using radiotherapy techniques that do not reflect modern‐day practice reported on disease‐free survival in women with low‐volume axillary disease (subdistribution HR 0.63, 95% CI 0.41 to 0.96, 1 study, 173 women). None of the included studies reported on PMRT side effects or quality‐of‐life outcome measures.

**Authors' conclusions:**

Based on one study, the use of PMRT in women diagnosed with breast cancer and low‐volume axillary disease indicated a reduction in locoregional recurrence and an improvement in survival. There is a need for more research to be conducted using modern‐day radiotherapy equipment and methods to support and supplement the review findings.

## Plain language summary

**Is X‐ray treatment (radiotherapy) after removal of breast tissue (mastectomy) better than no X‐ray treatment in women diagnosed with breast cancer that has spread to one to three armpit lymph nodes?**

**Key message:**

• Radiotherapy after breast removal (also known as mastectomy) may reduce cancer returning to the chest wall and in the nearby lymph nodes.

• Radiotherapy probably improves survival in women diagnosed with breast cancer who have 1 to 3 involved lymph nodes.

• We found one large ongoing international study that should provide more evidence when it has finished.

**What is breast cancer?**

Breast cancer is the most common form of cancer affecting women. The improvements in treatments (anticancer drugs) that target the entire body over the last 2 decades have resulted in fewer cancer‐related deaths. The development of recurrence in the chest wall and nearby lymph nodes after the initial treatment of breast cancer can increase the risk of cancer spreading. It has been shown that the risk of cancer recurring in the chest wall and nearby lymph nodes was more than doubled in women diagnosed with 1 to 3 positive axillary lymph nodes compared to node‐negative disease.

**What is the role of additional supportive treatment in the management of breast cancer?**

Radiotherapy is a well‐established supportive targeted treatment that reduces region‐specific recurrence after surgery in women diagnosed with breast cancer. However, researchers over the last 2 to 3 decades were not able to show without doubt that the reduction in recurrence central to a location with radiotherapy leads to fewer breast cancer‐related deaths.

**When should radiotherapy be used?**

Researchers agree about the use of radiotherapy after mastectomy in women with high‐risk breast cancer characteristics, like large tumour size (larger than 5 cm) and 4 or more axillary lymph nodes affected with breast cancer. However, scientists are still unsure about the role of radiotherapy after mastectomy in women with lower‐risk characteristics who are found to have 1 to 3 axillary lymph nodes affected by breast cancer.

**What did the review authors want to find out?**

For women diagnosed with breast cancer and found to have 1 to 3 lymph nodes affected by cancer, we asked:

• Does radiotherapy after breast removal reduce the occurrence of region‐based recurrence compared to no radiotherapy?

• Is there any survival advantage in women undergoing PMRT compared to those not having radiotherapy?

• What are the short‐ and long‐term unwanted effects of PMRT?

• Is there any difference in the quality of life for women undergoing radiotherapy compared to those with no radiotherapy?

**What did we do?**

We searched for studies that compared radiotherapy after mastectomy against no radiotherapy in women diagnosed with breast cancer with 1 to 3 involved axillary lymph nodes. Once we identified the relevant studies, we compared and summarised the results. We have also assessed and rated our confidence in the presented evidence based on the study methods and the number of women who participated in the studies.

**What did we find?**

We found 3 studies with 725 women; 355 women received radiotherapy, and 370 did not. The largest study was conducted in Denmark and involved 552 women. The remaining two studies were conducted in Sweden and involved 104 premenopausal and 173 postmenopausal women. Only the Danish study administered radiotherapy using methods that are comparable to modern‐day practice. All the studies followed the women for 15 years or more. The studies were funded by independent charitable organisations with no funding from private or pharmaceutical companies.

**Main results**

The use of radiotherapy compared to no radiotherapy in women diagnosed with breast cancer who have 1 to 3 involved axillary lymph nodes:

• may lead to a reduction in the regional recurrence; and

• probably improves survival by 24% in women.

We did not identify any study that reliably assessed the effect of radiotherapy after mastectomy on disease‐free survival, short‐ or long‐term unwanted effects and quality of life in breast cancer survivors.

**What are the limitations of the evidence?**

Our confidence in the evidence is low to moderate for 3 main reasons.

First, the studies we included took place before many modern advancements in breast cancer treatment. Second, we could only interpret results from one study that used updated radiotherapy techniques. Finally, the studies we used did not provide all of the information we wanted regarding the long‐term effectiveness of treatment in participants.

**How up to date is the evidence?**

The evidence presented here is up to date to September 2021.

## Summary of findings

**Summary of findings 1:** Summary of findings table ‐ Postmastectomy radiotherapy (PMRT) compared to no PMRT in women with breast cancer and one to three positive lymph nodes

**Postmastectomy radiotherapy (PMRT) compared to no PMRT in women with breast cancer and one to three positive lymph nodes**						
**Patient or population:** women with breast cancer and one to three positive lymph nodes **Setting:** Postoperative follow‐up **Intervention:** postmastectomy radiotherapy (PMRT) **Comparison:** no PMRT						
**Outcomes**	Anticipated absolute effects^*^ (95% CI)		**Relative effect (95% CI)**	**№ of participants (studies)**	**Certainty of the evidence (GRADE)**	**Comments**
	**Risk with no PMRT**	**Risk with postmastectomy radiotherapy (PMRT)**				
Local and regional recurrence (LRR) follow‐up: 15 years	10‐year risk of LRR		**HR 0.20** (0.13 to 0.33) [Local and regional recurrence]	552 (1 RCT)	⊕⊕⊝⊝ Low^b,^^c^	
	255 per 1000^a^	**57 per 1000** (38 to 93)				
Overall survival (OS) follow‐up: 25 years	10‐year risk of death		**HR 0.76** (0.60 to 0.97) [Overall survival]	552 (1 RCT)	⊕⊕⊕⊝ Moderate^b^	
	403 per 1000^a^	**324 per 1000** (266 to 394)				
Disease‐free survival (DFS)				(0 studies)	‐	No randomised controlled trials reported on disease‐free survival in PMRT women with low‐volume axillary disease.
Time to progression				(0 studies)	‐	No randomised controlled trials reported time to progression in PMRT women with low‐volume axillary disease.
Short‐term adverse events				(0 studies)	‐	No randomised controlled trials evaluated PMRT short‐term adverse events like erythema, hyperpigmentation and breast oedema.
Long‐term adverse events				(0 studies)	‐	No randomised controlled trials evaluated the long‐term adverse events of PMRT like lymphoedema, cardiac toxicity, pulmonary toxicity, bone necrosis and development of secondary radiation‐induced cancers.
Quality of life (QoL)				(0 studies)	‐	No randomised controlled trials measured or reported on the quality of life indicators after PMRT.
*The risk in the intervention group (and its 95% confidence interval) is based on the assumed risk in the comparison group and the **relative effect** of the intervention (and its 95% CI). **CI:** confidence interval; **HR:** hazard Ratio						
**GRADE Working Group grades of evidence** **High certainty:** we are very confident that the true effect lies close to that of the estimate of the effect. **Moderate certainty:** we are moderately confident in the effect estimate: the true effect is likely to be close to the estimate of the effect, but there is a possibility that it is substantially different. **Low certainty:** our confidence in the effect estimate is limited: the true effect may be substantially different from the estimate of the effect. **Very low certainty:** we have very little confidence in the effect estimate: the true effect is likely to be substantially different from the estimate of effect.						
See interactive version of this table: https://gdt.gradepro.org/presentations/#/isof/isof_question_revman_web_433754310228759346.						

^a^ Estimated from the no‐PMRT group published Kaplan‐Meier curve in one study (Overgaard 2007). ^b^ Downgraded quality of evidence by one level due to "serious concern about the risk of bias" originating from inadequate random sequence generation, lack of allocation concealment and not attempting to blind the assessors.  ^c^ Downgraded quality of evidence by one level due to the "serious imprecision" because of uncertainty in confidentially determining the spread of the observed effect in the PMRT group.

## Background

### Description of the condition

Breast cancer is the most common malignancy to affect women; in 2018, more than 2 million breast cancer cases were diagnosed globally, which accounted for 24.5% of all cancers in women (Bray 2018). The American Cancer Society has estimated that in 2020 around 276,480 new cases of invasive breast cancer will be diagnosed in the USA, with an estimated 42,690 deaths (Siegel 2020). Early diagnosis through screening and advancements in supportive (adjuvant) treatments over the years have resulted in improved outcomes for breast cancer survivors. This is reflected in the 1.5% average reduction in the age‐adjusted death rate per year between 2008 and 2017 in the USA compared to a 0.3% average rise of age‐adjusted new cases in the same period (SEER 2020).

The development of local and regional recurrence of breast cancer is a dreaded outcome that affects 5% to 15% of women diagnosed with breast cancer after mastectomy (removal of all breast tissue) and radiotherapy (EBCTCG 2014). Local and regional recurrence after primary breast cancer treatment is typically associated with an increased risk of concurrent and future spread of cancer elsewhere in the body (Van Tienhoven 1999). The 10‐year relative survival (cancer survival in the absence of other causes of death) after local and regional recurrence has been shown to be in the region of 25% to 50% even after attempts to remove the cancer recurrence (Chagpar 2003; Van Tienhoven 1999). The commonest area of local and regional recurrence after mastectomy is the chest wall (53%) followed by lymph nodes above and below the collar bone (26%) and in the armpit (13%) (Katz 2000; Nielsen 2006; Wallgren 2003). A palpable lump in these sites, painful enlarged lymph nodes and, in some cases, elevated red patches on the chest wall are common symptoms. The risk of local and regional recurrence after mastectomy substantially increases with the number of axillary lymph nodes containing breast cancer. The Early Breast Cancer Trialists' Collaborative Group (EBCTCG) individual participant data meta‐analysis demonstrated that the risk of local and regional recurrence is more than doubled in women who had a mastectomy with one to three lymph nodes affected with cancer compared to node‐negative women with breast cancer (EBCTCG 2005).

### Description of the intervention

Breast cancer is a systemic disease, and radiotherapy is a well‐established additional treatment (adjuvant) that aims to reduce local and regional recurrence (Fisher 1998; Fisher 2002). Radiotherapy involves radiation treatment using X‐rays or other forms of ionising radiation, and the doses are measured in Gray (Gy). Radiotherapy is administered in fractions to achieve maximum cancer control while attempting to reduce local complications. Up until April 2020, the standard practice in the UK for postmastectomy chest‐wall radiotherapy was 40 Gy in 15 fractions over a period of three weeks based on the evidence from the UK START A and B trials (START TRIAL A; START TRIAL B). Amongst women with node‐positive or high‐risk node‐negative breast cancer, the addition of regional nodal radiotherapy is dependent on risk stratification based on the characteristics of their cancer. The role of regional nodal irradiation has been evaluated in two large randomised controlled trials (RCTs), and both failed to identify any overall survival benefit but did show a significant reduction in breast cancer recurrence (Poortmans 2015; Whelan 2015). In contrast, a Danish population‐based cohort study showed an overall survival benefit of 4% for those women who received radiotherapy to lymph nodes near their breast bone (internal mammary) (Thorsen 2016). However, due to the lack of conclusive evidence of overall survival benefits in RCTs (Hennequin 2013; Poortmans 2015), regional nodal irradiation is recommended only in people with a high index of suspicion of nodal involvement or confirmed significant axillary nodal metastatic cancer (NICE 2018).

### How the intervention might work

Radiotherapy has been shown to reduce the 10‐year risk of local and regional recurrence by two‐thirds in women affected with breast cancer (EBCTCG 1995). However, the improvement in local and regional recurrence has not been shown to translate into a consistent survival advantage. The EBCTCG overview showed that for every 1.5 local and regional recurrences prevented in the first 10 years after radiotherapy, one breast cancer‐specific death could be prevented in 20 years (EBCTCG 2014). The absolute benefit in survival advantage might be much higher if we consider the advances in radiotherapy techniques over the last 20 years (EBCTCG 1995; Truong 2005). These include methods used to protect the heart, lungs and major blood vessels inside the chest wall from radiotherapy‐induced morbidity and mortality in women with breast cancer. The findings of the EBCTCG overview (EBCTCG 2000) and long‐term outcomes of some seminal RCTs (Overgaard 2007; Ragaz 2005) were considered by the St Gallen Consensus Guidelines (2009), which recommended postmastectomy radiotherapy (PMRT) for women affected with breast cancer who had a 20% or greater 10‐year risk of local and regional recurrence (Goldhirsch 2009).

### Why it is important to do this review

Postmastectomy radiotherapy (PMRT) is currently recommended for women with breast cancer who have four or more lymph nodes involved with metastatic cancer. There is still no international consensus on whether to offer PMRT to women with breast cancer and one to three axillary lymph nodes affected with cancer (low‐volume axillary metastatic disease). The National Comprehensive Cancer Network (NCCN) guidelines recommend giving strong consideration to providing PMRT to women with one to three metastatic axillary lymph nodes (Salerno 2017). However, there is currently no risk stratification model based on demographic or cancer characteristics to enable clinicians to make these difficult choices and decisions. Age, location of the tumour in the inner aspect of the breast, nodal ratio (i.e. the number of lymph nodes with cancer versus the total number of lymph nodes removed during axillary surgery), lymphovascular invasion (cancer invading the small blood vessels and lymphatics in the breast tissue) and oestrogen receptor negativity (i.e. an absence of female hormone molecule binding sites on the surface of cancer cells), tumour size and positive resection margins (Garg 2007; Truong 2005) are some of the variables that have been shown to be predictors of local and regional recurrence in women with PMRT with low‐volume axillary nodal disease. Predictive tools such as the Cambridge PMRT index have been developed to try and select women who benefit from radiotherapy in this setting (Mukesh 2014 ). The predictive role of age was considered by both the NCCN and the European Society of Breast Cancer Specialists, and they recommend PMRT in young women affected with breast cancer and metastasis in one to three axillary lymph nodes (Cardoso 2012; Recht 2016; Salerno 2017). Similarly, the St Gallen breast cancer meeting in 2019 recommended PMRT in triple‐negative breast cancer (i.e. where there is a lack of expression of oestrogen, progesterone and human epidermal growth factor receptor‐2 protein molecule binding sites on the surface of cancer cells) with one to three positive axillary lymph nodes (Burstein 2019).

The Selective Use of Postoperative Radiotherapy aftEr MastectOmy (SUPREMO) Trial is the only ongoing RCT that might shed light on the role of modern adjuvant systemic treatment (antihormonal tablets or chemotherapy) in PMRT for women diagnosed with breast cancer and found to have metastasis in one to three lymph nodes. Since the result of this study is not expected to be published before 2024, this review will try to bridge the gap in the literature by evaluating the long‐term outcomes of published RCTs to address the uncertainty surrounding the use of PMRT in women with breast cancer who have a low volume of axillary lymph node disease.

## Objectives

To assess the effects of PMRT in women diagnosed with early breast cancer and found to have one to three positive axillary lymph nodes.

## Methods

### Criteria for considering studies for this review

#### Types of studies

Randomised controlled trials (RCTs) evaluating PMRT in women diagnosed with early breast cancer and low‐volume axillary metastatic disease, which is defined as the involvement of cancer cells in one to three axillary lymph nodes after sentinel lymph node biopsy (SLNB) or axillary lymph node dissection (ALND).

#### Types of participants

We included women diagnosed with early breast cancer and found to have one to three positive axillary lymph nodes. This included women who were found to have a macroscopic (≧ 2 mm in size) deposit of cancer in the axillary lymph nodes (macrometastases). We contacted the corresponding study authors to obtain data on macrometastasis if data were missing or described only as part of a subgroup analysis within the published manuscripts.

We included women diagnosed with breast cancer treated with mastectomy and SLNB without any further axillary surgery as well as those who were undergoing ALND with or without initial SLNB. We included women who were undergoing either simple or modified radical mastectomy, while we excluded those undergoing radical mastectomy. To improve the generalisability of the results, we included women of all ages and ethnicities, who have been diagnosed with breast cancer, with any tumour size(s) and with any histological types.

#### Types of interventions

We included only studies in which radiotherapy was given using X‐rays (photon radiation) and electrons. The total radiation dose administered for treatment should be consistent with the current recommendations (i.e. 40 Gy to 50 Gy in 15 to 25/28 fractions over 3 to 5 weeks). We included studies in which PMRT was given to the ipsilateral chest wall, axilla, supraclavicular fossa, and internal mammary nodes. We excluded studies in which women diagnosed with breast cancer received only intraoperative radiation, brachytherapy or radiotherapy given using gamma rays.

We included studies where adjuvant treatments (endocrine, chemotherapy and biological agents) were given to both the intervention and comparison groups. While we included women who were given chemotherapy after mastectomy (adjuvant chemotherapy), we excluded studies that used neoadjuvant chemotherapy, where chemotherapy was given before surgery. Neoadjuvant chemotherapy was usually offered to women diagnosed with large breast cancers and those with multiple positive axillary lymph nodes. It was given with the intention of reducing the size of cancer to facilitate breast‐conserving surgery and aid in the decision‐making of postsurgical adjuvant treatment. In most cases, progression of the disease or poor response to neoadjuvant chemotherapy was the commonest reason for offering mastectomy before or after completion of neoadjuvant chemotherapy. Finally, the pre‐neoadjuvant chemotherapy cancer characteristics mainly guide the decision to give adjuvant radiotherapy to these women with breast cancer. All these factors detract from the main focus of this review, hence we have excluded studies where neoadjuvant chemotherapy was administered.

We have compared PMRT in women with early breast cancer with the low‐volume axillary disease to those who did not receive any radiotherapy.

#### Types of outcome measures

##### Primary outcomes

- Local and regional recurrence is defined as the duration in years between the treatment of breast cancer and recurrence of breast cancer in the ipsilateral chest wall, axilla, supraclavicular fossa, infraclavicular fossa or internal mammary nodes. We considered local and regional recurrence as the first event after treatment of breast cancer and thereby prior to the development of any systemic recurrence (i.e. recurrence of breast cancer anywhere else in the body other than those sites involved by local and regional recurrence). In this review, when it was not possible to extract results for the time to local and regional recurrence, we attempted to extract the percentage of women who had a local and regional recurrence at 5, 10 and 15 years.

##### Secondary outcomes

- Overall survival: defined as the duration between the diagnosis of breast cancer or the date of surgery to the date of death from any cause
- Disease‐free survival: defined as the duration between the diagnosis of breast cancer or time of surgery to the date of locoregional or systemic recurrence or death, whichever occurs first
- Time to progression: defined as the duration between the diagnosis of breast cancer or time of surgery to the date of locoregional or systemic recurrence, whichever occurs first
- Adverse events, including short‐ and long‐term events: Short‐term adverse events will include erythema, hyperpigmentation and breast oedema. Long‐term adverse events will include lymphoedema, cardiac toxicity, pulmonary toxicity, bone necrosis and the development of secondary radiation‐induced cancers.
- Quality of life: measured using any validated tool(s)

### Search methods for identification of studies

#### Electronic searches

We searched the following databases and registries on 24 September 2021:

- The Cochrane Breast Cancer Group's (CBCG's) Specialised Register. Details of the search strategies used by the Group for the identification of studies and the procedure used to code references are outlined in the Group's module (https://breastcancer.cochrane.org/specialised-register). Studies with the keywords "breast cancer”, “mastectomy”, “radiotherapy”, “radiation therapy”, “post‐operative radiotherapy” and “post‐mastectomy radiotherapy” were extracted and considered for inclusion in the review.
- Cochrane Central Register of Controlled Trials (CENTRAL; 2021, Issue 9) in the Cochrane Library (searched 24 September 2021). See Appendix 1.
- MEDLINE Ovid (1946 to 24 September 2021). See Appendix 2.
- Embase Ovid (1947 to 24 September 2021). See Appendix 3.
- The WHO International Clinical Trials Registry Platform (ICTRP) search portal (http://apps.who.int/trialsearch/Default.aspx) for all prospectively registered and ongoing studies. See Appendix 4.
- Clinicaltrials.gov (http://clinicaltrials.gov/). See Appendix 5.

#### Searching other resources

##### Bibliographic searching

We reviewed the reference lists of the included studies or reviews identified through the search.

### Data collection and analysis

#### Selection of studies

Two review authors (RV and MC) looked over the studies identified from the search strategy independently. Each review author applied the selection criteria to identify relevant studies for inclusion. If the review authors disagreed, they reached a consensus through deliberation with the help of a third review author (SSR). We used the PRISMA flow diagram (Page 2021) to describe the selection process and recorded all excluded studies in the characteristics of the excluded studies table (Figure 1). We did not apply any restrictions regarding the language or publication date of the studies.

**1:**
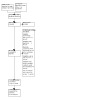
PRISMA flow diagram

#### Data extraction and management

Two review authors (RV and MC) extracted the data, and they resolved any disagreements through discussion with JB and SSR. We collected available data on demographics (age), tumour characteristics (tumour size, grade and receptor status), adjuvant treatments and outcome measures. We performed pooled statistical analysis on outcome measures when there were sufficient data available from the included studies.

We entered data into RevMan Web for analysis. We requested further information from the corresponding study authors as required about the statistical methods, analysis and results. In circumstances where corresponding study authors did not provide the data that we requested, we have attempted to extract the necessary information from the published results using well‐established statistical methods (Tierney 2007).

#### Assessment of risk of bias in included studies

Two review authors (RV and MC) assessed the risk of bias independently for each of the included studies, and they resolved any disagreement by discussion with the corresponding review author (SSR). We used Cochrane's risk of bias tool (RoB 1; Higgins 2011). This tool involves seven domains to address the quality of randomisation and the degree of bias arising in an RCT. Each domain was divided into three categories ‐ 'low', 'unclear' or 'high' risk ‐ on the basis of specific criteria described in the tool. These judgements enabled us to categorise studies on the basis of their risk of bias and to perform sensitivity analysis when required to assess the effect of the quality of the included studies on the results.

#### Measures of treatment effect

We reported on time‐to‐event outcome measures (i.e. local and regional recurrence, overall survival and disease‐free survival) as hazard ratios (HRs) with 95% confidence intervals (CIs). We extracted the data indirectly from reported results (e.g. log‐rank P values) or Kaplan‐Meier survival curves (Parmar 1998; Tierney 2007) when the HR and associated variance were not reported in the published literature.

We reported dichotomous outcomes as risk ratios (RRs) with 95% CIs. RR values less than 1 indicate that PMRT is the better treatment option, while RR values greater than 1 indicate that no radiotherapy after mastectomy is better for women with breast cancer and low‐volume axillary disease.

For future review versions, if continuous outcome measures (i.e. quality of life) are reported, we will use the standardised mean difference and 95% CI where different scales are used to measure quality of life across studies. If similar scales are used to measure quality of life, we will report the mean difference.

#### Unit of analysis issues

The unit of analysis was each individual woman diagnosed with breast cancer. Since we were comparing the effect of PMRT against no radiotherapy in low‐volume axillary disease, we did not consider cross‐over studies and multiple intervention groups. Multiple events per woman were considered only when the second event was a different outcome measure (i.e. if a person develops local and regional recurrence as the first event and then dies due to breast cancer or any other cause (disease‐free survival or overall survival)).

#### Dealing with missing data

There was minimal attrition bias among the included studies in this review. All the studies identified as being eligible for inclusion were subgroup analyses of original RCTs performed to address the effectiveness of PMRT in women diagnosed with breast cancer.

#### Assessment of heterogeneity

We used Cochran's Q test and the I^2^ statistic for assessing statistical heterogeneity (Cochran 1954; Higgins 2003). For the I^2^ statistic, a value of 25% to 50% may represent mild statistical heterogeneity, 50% to 75% may represent moderate statistical heterogeneity, and > 75% may represent considerable statistical heterogeneity (Deeks 2022). We used a random‐effects model for analysis when there was clinical heterogeneity (i.e. variation in women, interventions or outcomes) or methodological heterogeneity (i.e. variation in study design, outcome measurement or risk of bias) in the included studies.

#### Assessment of reporting biases

We could not assess for publication bias using a funnel plot because we included fewer than 10 eligible studies in the review as recommended by the *Cochrane Handbook of Systematic Reviews of Interventions* (Page 2022).

#### Data synthesis

We performed data synthesis and statistical analysis in RevMan Web software. We used random‐effects models that employ the DerSimonian and Laird method (DerSimonian 1986), as the I^2^ statistic showed moderate to substantial statistical heterogeneity. Some of the outcome measures were represented narratively due to the lack of sufficient studies to pool the results or when it was not reported in any of the included studies.

#### Subgroup analysis and investigation of heterogeneity

We failed to identify any studies investigating the role of age, type of axillary surgery (SLNB alone versus ALND), oestrogen receptor and human epidermal growth factor‐2 receptor (Her2) status on local and regional recurrence in women with early breast cancer and low‐volume axillary nodal disease treated with or without PMRT. Hence, we did not perform any subgroup analysis within an individual study or across included studies.

#### Sensitivity analysis

We did not perform a sensitivity analysis as we only identified three studies, and we describe the outcome from a single study pertaining to modern‐day radiotherapy practice in this review.

#### Summary of findings and assessment of the certainty of the evidence

Two review authors (RV and MC) assessed the overall certainty of the evidence by using the GRADE approach (Schünemann 2022). This involved assessing the evidence for each outcome measure using five domains. These domains relate to the risk of bias in the included studies, inconsistency, indirectness, imprecision and publication bias. We created a summary of findings table to address each of these domains for the included studies using GRADEpro GDT software for primary and secondary outcome measures.

All the included studies in this review were subgroup analyses of the original randomised cohort of women diagnosed with breast cancer and treated with PMRT. Hence none of the included studies was statistically powered to determine the desired or reported outcome measures. Moreover, the type and duration of adjuvant systemic treatments used in the studies included in this review were suboptimal compared to the current standard of care.

The study authors of the South Sweden Breast Cancer Group (SSBCG) studies have acknowledged that a lack of a primary hypothesis with no distinction between primary and secondary outcome measures could have resulted in a sample size calculation that was inadequate (Killander 2009). This was compounded by the fact that they failed to accrue enough women (150 women in each of the three arms of the study) as per the proposed sample size calculation (Killander 2009). In both studies, there was a gap of 3 weeks between the first 12 doses of radiotherapy and the last 8 doses. This is not a standard practice in the modern‐day administration of radiotherapy following mastectomy.

## Results

### Description of studies

#### Results of the search

We retrieved 5328 records from medical databases (CENTRAL, Medline, Embase and CBCG's Specialised Register) and 354 records from clinical study registries (ClinicalTrials.gov and WHO ICTRP). After the removal of duplicate records, we screened 5461 records. Since the review was specifically looking at RCTs, we used the RCT classifier to help distinguish between RCTs (3400 records; high‐probability group) and non‐RCTs (1928 records; low‐probability group). Two review authors (RV and MC) screened the high‐probability group for eligible studies and achieved consensus through discussion with the third review author (SSR). One review author (SSR) screened the low‐probability group and discussed any uncertainties with a second review author (RV). We identified 25 records for full‐text or further review from the high‐probability group and none from the low‐probability group. We selected three studies to be included and one ongoing study for the review and excluded 21 studies with the reasons described in the PRISMA flow chart and Characteristics of excluded studies. See Figure 1.

#### Included studies

Three studies that fulfilled the eligibility criteria for this review originated from two of the largest randomised studies undertaken in the early 1980s to address the role of PMRT in adjuvant settings (Killander 2007; Killander 2009; Overgaard 2007). Of these three studies, one study used radiotherapy techniques that are directly comparable to modern‐day practice (Overgaard 2007).

Overgaard 2007 was a subgroup analysis undertaken from the original Danish Breast Cancer Cooperative Group (DBCG) studies investigating the role of PMRT in addition to adjuvant systemic therapy in premenopausal (DBCG 82b: Overgaard 1997) and postmenopausal (DBCG 82c: Overgaard 1999) women diagnosed with breast cancer. The subgroup analysis involved only those randomised women with eight or more lymph nodes removed during axillary node clearance and then found to have one to three lymph nodes involved with breast cancer (Overgaard 2007). Participants were randomised to PMRT (n = 256) or no PMRT (n = 256) after total mastectomy and removal of level 1 and partly level 2 axillary lymph nodes. The radiotherapy was administered using a linear accelerator with a target volume of 50 Gy in 25 fractions over 35 days or 48 Gy in 22 fractions over 38 days. Systemic adjuvant therapy was offered based on the menopausal status. All the premenopausal and menopausal women received eight to nine cycles of cyclophosphamide, methotrexate and 5‐fluorouracil (CMF regimen) intravenously every four weeks for nine months. Whereas, postmenopausal women received oral tamoxifen 30 mg daily for 48 weeks. The outcome measures reported in this study included both the locoregional recurrence and overall survival.

In their randomised study, the SSBCG published their outcome separately for premenopausal women (Killander 2009) and postmenopausal women (Killander 2007). The data specific to women diagnosed with breast cancer and found to have one to three involved lymph nodes were available in both the above studies published by the SSBCG. In Killander 2009 and Killander 2007, either premenopausal or postmenopausal women diagnosed with breast cancer and treated with modified radical mastectomy and axillary node clearance were randomised. The randomisation was stratified to the department, tumour size and the number of positive axillary lymph nodes. The study participants were randomised in a 1:1:1 fashion to receive radiotherapy alone, radiotherapy with adjuvant systemic treatment (PMRT group) and only adjuvant systemic treatment (no‐PMRT group). We did not include the radiotherapy‐alone group in this review as the group did not receive any adjuvant systemic treatment and hence were not comparable to the other two groups (i.e. PMRT and no PMRT). The radiotherapy was administered using an orthovoltage ventral beam or an electron ventral field technique to the chest wall with megavoltage photons to the lymph node regions. There was a three‐week gap between the first 12 doses of radiotherapy and the last 8 doses. The maximum targeted skin dose of 45 Gy was administered in 20 fractions over a period of 5 weeks. The treatment protocol with a three‐week gap to diminish skin reactions on the chest wall resulted in a large reduction in the total effective treatment dose. Haviland 2016 estimated that in breast cancer 0.6 Gy per day may be lost in the gap when using regimens of 2 Gy per fraction. The 1.9 Gy per fraction used in this protocol is very close to 2 Gy, and therefore it is estimated that as much as 12.6 Gy of dose could be lost in a three‐week gap during treatment.

Killander 2007 (postmenopausal group) involved women randomised to receive PMRT (n = 79) and no PMRT (n = 94). Both groups were given tamoxifen (30 mg/day) for one year as adjuvant treatment. They reported on local and regional recurrence, time to systemic disease (a surrogate marker for disease‐free survival) and overall mortality without any distinction between primary and secondary outcome measures.

Killander 2009 (premenopausal group) involved women randomised to receive PMRT (n = 54) or no PMRT (n = 50). Both groups were given 12 cycles of oral cyclophosphamide as adjuvant chemotherapy every 4 weeks. The outcome measures considered were local and regional recurrence with the occurrence of systemic disease and death being competing events, time to systemic disease (a surrogate marker for disease‐free survival) with the occurrence of non‐breast cancer death as a competing event and overall mortality. The results were described using cumulative incidence and mortality curves. There was insufficient information provided to extract the HR for the outcome measures using the methods described by Tierney et al (Tierney 2007) in this study. As per Killander 2007, there was no distinction between primary and secondary outcome measures in this study.

##### Ongoing studies

We identified one ongoing study in follow‐up phase, and the results are expected in early 2024 (SUPREMO Trial ‐ Velikova 2018). The SUPREMO study is an open‐label, parallel‐group RCT that has finished recruiting but is in the active phase of follow‐up. Eligible participants were women aged 18 years or older undergoing mastectomy for unilateral intermediate‐risk breast cancer. The intermediate risk was defined as those women with a tumour size less than 5 cm and one to three involved lymph nodes as well as those that are node‐negative with tumour size more than 2 cm but does not involve the skin or underlying muscle. There was only a single study that specifically addressed the quality of life outcome after PMRT (Velikova 2018).

The SUPREMO quality of life substudy was preplanned and aimed to examine the effect of PMRT on several quality of life outcome measures at 1, 2, 5 and 10 years only from women recruited in the UK. They published their preliminary results of the quality of life outcome after two years of follow‐up (Velikova 2018). However, the published results included all participants randomised, including women with no involved lymph nodes after axillary staging surgery (N0 disease). Since the study is still ongoing, the study authors were unable to release the data specific to women with one to three involved lymph nodes (N1 disease).

#### Excluded studies

We excluded 21 studies after full‐text review (Arriagada 1995; Baum 1980; Cahlon 2015; Friedl 1983; Gustavsson 1999; Højris 1999; Højris 2000; Houghton 1994; Killander 2014; Morgan 2002; Nielsen 2006; Osman 2014; Overgaard 2020; Ragaz 2005; Rutqvist 1990; Rutqvist 1992; Rutqvist 2006; Schmoor 2000; Seegenschmiedt 2000; Shi 2011; Smith 2001). The reasons for exclusions are detailed in the Characteristics of excluded studies tables.

### Risk of bias in included studies

See Figure 2.

**2:**
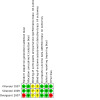
Risk of bias summary for the included studies

#### Allocation

Two studies used adequate methods to generate a random sequence and centralised stratified randomisation process (Killander 2007; Killander 2009) and were judged as low risk of bias.

One study (Overgaard 2007), which was a subgroup analysis of randomised women with one to three lymph nodes, selected only those women with eight or more lymph nodes removed during the axillary node clearance. This was done to increase the internal and external validity of the subgroup study as the original randomised cohort of the DBCG 82b (Overgaard 1997) and 82c (Overgaard 1999) studies had only a median of seven lymph nodes removed during the axillary nodal clearance. The study authors have not clearly stated how many randomised women with one to three lymph nodes were excluded in this subgroup analysis, thereby introducing selection bias. Thus the study was assessed as having an inadequate randomisation method and judged as high risk of bias in this domain. The same study did use closed‐envelope randomisation at each of the recruiting departments (Overgaard 2007) and was assessed as having adequate allocation concealment.

#### Blinding

Blinding of neither the women nor those administering the treatment was possible due to the use of radiotherapy as the primary intervention. We were not able to identify any description of blinding being considered or attempted in the published manuscript of the included studies.

A lack of blinding of women and personnel and when assessing outcomes was not considered to be a serious concern of bias given the objective nature of the outcomes reported. All three studies were deemed at unclear risk of bias for performance and detection bias.

#### Incomplete outcome data

The studies included in this review reported on a subgroup of women identified to have one to three involved lymph nodes amongst the entire randomised cohort. The use of ongoing follow‐up data, revisiting individual hospital records and the national population registry maintained in Denmark and South Sweden ensured near‐complete follow‐up data of the included women. Hence in this review, none of the included studies reported a dropout rate of more than 10%, thus minimising the attrition bias (Killander 2007; Killander 2009; Overgaard 2007).

#### Selective reporting

The included studies were all registered with the clinical study registries, and all the relevant outcome measures were reported as described in the study protocol. Thus all three studies were deemed at low risk of bias for selective reporting of outcomes.

However, it should be emphasised that all the studies were originally recruited to evaluate the role of PMRT in women diagnosed with breast cancer. Hence evaluating the role of PMRT in a subgroup of women with low‐volume axillary disease was unplanned.

#### Other potential sources of bias

In a very small proportion of women (n = 133; 4.3%) in the entire randomised cohort (n = 3078) of the DBCG 82b (Overgaard 1997) and 82c studies (Overgaard 1999), orthovoltage radiotherapy was used instead of a linear accelerator to deliver electrons/photons. The lowest intended dose was 36 Gy in 20 fractions given over 4 weeks using the McWhirter technique (Overgaard 1999). However, it was not possible to ascertain how many of these women were part of the subgroup analysis of 552 women with one to three positive lymph nodes (Overgaard 2007) relevant to this review. Given the small number of randomised women to whom orthovoltage radiotherapy was given, we did not consider that it would influence the outcome measures considered in this review.

In Killander 2007 and Killander 2009, radiotherapy was administered using two different techniques to the chest wall and lymph nodal regions. Moreover, there was a 3‐week gap between the 12thdose and the final 8 doses of radiotherapy. This resulted in a reduction of 12.6 Gy biological equivalent dose delivered to the chest wall and nodal regions. Hence the outcome from both these studies was not pooled for performing a meta‐analysis and has been explained in a descriptive manner.

The adjuvant treatment in all the included studies was suboptimal compared to current standard practice. In postmenopausal women, tamoxifen was given (30 mg/day) for one year only (Killander 2007; Killander 2009; Overgaard 2007) compared to the current standard practice of a minimum of five years of adjuvant endocrine treatment. Moreover, in postmenopausal women, tamoxifen was given as adjuvant systemic treatment irrespective of the oestrogen receptor status (Killander 2007).

The use of adjuvant systemic chemotherapy has evolved and is significantly different from the agents used in the included studies. Killander 2009 used 12 courses of oral cyclophosphamide 130 mg/m^2^ for 14 days every 4 weeks, while Overgaard 2007 used 8 to 9 cycles of CMF (cyclophosphamide 600 mg/m^2^, methotrexate 40 mg/m^2^ and 5‐fluorouracil 600 mg/m^2^) intravenously every 4 weeks for 9 months. These differences in practice should be considered while interpreting the results of this review.

### Effects of interventions

See: Table 1

#### Local and regional recurrence

The primary outcome measure was described in one study, which used radiotherapy techniques comparable to modern‐day radiotherapy methods (Overgaard 2007). There was a substantial reduction in local and regional recurrence in the PMRT group (HR 0.20, 95% CI 0.13 to 0.33, 522 women; low‐certainty evidence; Analysis 1.1).

In the remaining two studies (Killander 2007; Killander 2009), there was insufficient information provided to extract HRs for local and regional recurrence, and we were unable to obtain the individual data from the study authors. However, cumulative incidence at 20 years, accounting for the competing risks of distant recurrence and death, was reported in both studies. Both studies (Killander 2007; Killander 2009) reported decreased cumulative incidence of local and regional recurrence at 20 years in the PMRT group compared to the no‐PMRT group. In postmenopausal women, the cumulative incidence of local and regional recurrence was 2.6% (95% CI 0.5 to 8.3, 79 women) in the PMRT group and 25.9% (95% CI 17.5 to 35.1, 94 women) in the no‐PMRT group (Killander 2007). In premenopausal women, the cumulative incidence of local and regional recurrence was 3.9% (95% CI 0.7 to 11.9, 54 women) and 14.8% (95% CI 6.5 to 26.3, 50 women), respectively (Killander 2009). Even though we were unable to extract the HR from these two studies, there was concordance in the findings, suggesting a reduction in local and regional recurrence amongst the PMRT group compared to the no‐PMRT group.

#### Overall survival

The overall survival was reported in two studies at 15 years (Overgaard 2007) and 25 years (Killander 2007) follow‐up. The one study that used radiotherapy techniques comparable to modern‐day radiotherapy methods reported an improvement in overall survival amongst the PMRT group compared to the no‐PMRT group (HR 0.76, 95% CI 0.60 to 0.97, 552 women; moderate‐certainty evidence; Analysis 1.2; Overgaard 2007).

In the other study involving postmenopausal women (Killander 2007), there was little difference in overall survival amongst the PMRT group compared to the no‐PMRT group (HR 0.90, 95% CI 0.66 to 1.20, 173 women).

It was not possible to extract HRs for the study involving premenopausal women (Killander 2009), but overall mortality at 20 years was reported in the PMRT group as 33% (95% CI 25 to 48) compared to the no‐PMRT group at 50% (95% CI 38 to 64) and did not reach statistical significance (P = 0.086).

#### Disease‐free survival

Data for disease‐free survival were not reported by the one study using radiotherapy techniques comparable to modern radiotherapy methods (Overgaard 2007).

The remaining studies reported some disease‐free survival data. Killander 2007 reported the outcome in a cumulative incidence curve, accounting for the competing risk of non‐breast cancer deaths. Applying the methods described by Tierney 2007, a subdistribution HR was extracted comparing the cumulative incidence of disease‐free survival in the PMRT group compared to the no‐PMRT group (Fine 1999). The subdistribution HR should be interpreted in the context of a competing risks analysis, where the impact of factors affecting the competing risk on the cumulative incidence of disease‐free survival was taken into account (McCaw 2022). The subdistribution HR extracted from the cumulative incidence curves reported by Killander 2007 showed a statistically significant improvement in disease‐free survival amongst the PMRT group (subdistribution HR 0.63, 95% CI 0.41 to 0.96, 173 women; Analysis 1.3).

Killander 2009 provided the cumulative incidence of disease‐free survival at 20 years in the premenopausal group and reported no difference in the cumulative incidence of systemic disease between the PMRT group (35%; 95% CI 22 to 48) and no‐PMRT group (38%; 95% CI 24 to 51).

#### Time to progression

The studies did not report this outcome for women diagnosed with breast cancer and one to three positive lymph nodes and randomised to have PMRT.

#### Short‐term adverse events

The studies did not report this outcome for women diagnosed with breast cancer and one to three positive lymph nodes and randomised to have PMRT.

#### Long‐term adverse events

The studies did not report this outcome for women diagnosed with breast cancer and one to three positive lymph nodes and randomised to have PMRT.

#### Quality of life

We identified one ongoing study (SUPREMO) that reported on the two‐year quality of life outcome after PMRT (Velikova 2018). However, since the study is in the active follow‐up stage, the study authors could not release the data specific to women diagnosed with one to three involved lymph nodes. Hence, we were unable to report on this important outcome measure in the current review.

## Discussion

### Summary of main results

Three RCTs met the inclusion criteria (Killander 2007; Killander 2009; Overgaard 2007), with only one study using radiotherapy techniques that are comparable to modern‐day practice (Overgaard 2007). All studies were all subgroup analyses of original RCTs conducted in the 1980s to assess the effectiveness of PMRT in women diagnosed with breast cancer. Hence, the type and duration of adjuvant systemic treatments used in the included studies were suboptimal compared to the current standard of care.

The one study that most reflected current radiotherapy practice (Overgaard 2007) reported local and regional recurrence and overall survival. Low‐certainty evidence indicated an improvement in local and regional recurrence. Moderate‐certainty evidence showed an improvement in overall survival amongst the PMRT group compared to the no‐PMRT group.

One of the other studies that did not use modern‐day radiotherapy techniques reported disease‐free survival and showed a significantly better 20‐year disease‐free survival in the PMRT group than the no‐PMRT group (Killander 2007).

The lack of published evidence meant that we were not able to report on the time to progression, adverse events and quality of life secondary outcomes in this review. A recent study has reported on quality of life outcomes after PMRT (Velikova 2018) as a substudy of the ongoing SUPREMO Trial. However, the study investigators were unable to provide quality of life data specifically for women diagnosed with one to three positive lymph nodes due to the ongoing nature of the SUPREMO Trial.

### Overall completeness and applicability of evidence

There is a paucity of data on this topic. The results presented in this review were drawn from 3 RCTs. We were not able to pool the results to perform a meta‐analysis as only one study pertained to the modern‐day radiotherapy technique. The result for the primary endpoint of local and regional recurrence was extracted from a single RCT. We were not able to identify any studies to address the secondary outcome measures related to time to progression, short‐ and long‐term side effects and quality of life after PMRT.

The included studies were all subgroup analyses of previously conducted RCTs performed to evaluate the role of PMRT in women diagnosed with breast cancer. Hence, they were not designed specifically to answer the effectiveness of PMRT in women with breast cancer and one to three positive lymph nodes.

There is a lack of evidence within the literature on the effectiveness of the current standard‐of‐care radiotherapy techniques in women with breast cancer and low‐volume axillary disease. The radiotherapy in all included studies was suboptimal compared to the current standard practice with modern photon radiotherapy. None of the studies reviewed used megavoltage photons as the primary treatment for the chest wall target area. The use of intensity‐modulated photon radiotherapy, volumetric modulated arc therapy and deep inspiratory breath hold radiotherapy was not standard practice at the time of the included studies in this review. These modern radiotherapy techniques can improve the therapeutic ratio between cancer outcomes and late toxicity. Due to the lack of data on modern radiotherapy techniques in the low‐volume axillary disease group, we limited our inclusion of radiotherapy intervention to those techniques considered acceptable to the current ongoing SUPREMO Trial so that future comparisons could be made and excluded older interventions such as cobalt‐60 radiotherapy.

Further consideration should be given while interpreting the results of this review in light of the type of systemic endocrine and chemotherapy medications used in the included studies. The advancement in modern endocrine, chemotherapy and targeted immunotherapy treatments may mitigate or reduce the magnitude of the improvement in overall survival observed with PMRT in this review.

There was a lack of published and available data to evaluate the role of dose escalation, uses of chest wall bolus and chest wall boost as part of the PMRT technique in this review.

### Quality of the evidence

We identified three RCTs that fulfilled the inclusion criteria involving a total of 829 women (PMRT 409, no PMRT 420). We were unable to extract data from one of the included studies (Killander 2009) to pool the outcome measures and hence described the findings in a descriptive manner (PMRT 54, no PMRT 50).

The internal validity of one of the largest studies (Overgaard 2007) was compromised due to the method used for randomisation and allocation concealment. The studies included in this review were all subgroup analyses of original RCTs conducted in the early 1980s to evaluate the role of PMRT in breast cancer. This allowed the included studies to be comparable and hence reduced statistical heterogeneity. However, the type and duration of various systemic adjuvant treatments being offered to the women randomised in these studies were substantially different in efficacy compared to the modern chemo‐endocrine treatments. There was a very low attrition bias at the expense of compromised power due to the subgroup analysis performed in the included studies.

The overall confidence in the primary outcome measure (local and regional recurrence) is low due to the inability to pool the data and the concerns about the quality of the evidence of the only study from which the data were extracted. The estimated effect seen for overall survival is more likely to be closer to the expected true effect.

### Potential biases in the review process

Since this was an interventional review, it was essential to include only RCTs to ensure that both PMRT and no‐PMRT groups remain comparable. Even though this minimised selection bias, it resulted in the identification of only three RCTs eligible for inclusion in this review. We wanted to ensure that the type of radiotherapy used in the included studies was comparable to what would be acceptable as close to the current standard practice as possible and therefore accepted techniques that would be included in the ongoing SUPREMO Trial (Velikova 2018). Hence, amongst three eligible RCTs, only a single study pertaining to the modern‐day radiotherapy technique was represented in the main outcome measures to allow us to combine the data once the results of the SUPREMO Trial are published. This also limited the number of studies that could be included in this review compared to some other larger reviews and meta‐analyses that have been published (EBCTCG 2014; Li 2013; Whelan 2000). There are very few studies that have reported short‐term side effects after PMRT, and we were unable to identify any studies reporting long‐term side effects. This was potentially due to two factors: lack of side effects reported specifically in the one to three positive lymph node groups and lack of predefined follow‐up of RCTs to ascertain the long‐term side effects.

We identified two additional studies that could have been included in this review. However, the lack of information in the published report and the inability to gather information from the study authors meant that it was not possible to ascertain whether these studies were randomised, quasi‐randomised or prospective cohort studies (Osman 2014; Shi 2011). We were unable to combine the data for performing a pooled analysis for our primary and secondary outcome measures. This was due to only one out of the three eligible studies using radiotherapy techniques that are comparable to modern‐day methods. There were also different statistical methods used for reporting the secondary outcome measures in the eligible studies for inclusion with some studies reporting insufficient information to extract HRs. Finally, we were unable to obtain any data on the side effects and quality of life outcomes after PMRT. Both of these outcome measures are important in helping to understand the overall effect of PMRT in women with one to three positive lymph nodes.

### Agreements and disagreements with other studies or reviews

There are four published meta‐analyses evaluating the role of PMRT in women with one to three positive lymph nodes (EBCTCG 2014; Headon 2016; Li 2013; Whelan 2000).

Whelan 2000 presented one of the earliest meta‐analyses evaluating the role of PMRT and included 18 randomised studies between 1967 and 1999, involving 6367 women diagnosed with breast cancer. They included all studies in which PMRT was administered irrespective of the axillary lymph nodal status. In spite of this, they were able to demonstrate an overall survival benefit for women undergoing PMRT. Li 2013 performed a meta‐analysis of nonrandomised cohort studies reporting on the role of PMRT in one to three positive nodes. They were able to show that there was a substantial improvement in local and regional recurrence with PMRT (degrees of freedom (df) 9, RR 0.348, 95% CI 0.254 to 0.477) from the 10 studies eligible for inclusion. However, the pooled analysis from six eligible studies showed no improvement in overall survival amongst women receiving PMRT. However, it should be noted that most of the women in these studies were treated between 1983 and 2006, resulting in substantial variability in the adjuvant treatments being offered, which would have an effect on the reported outcome measures.

The EBCTCG 2014 published an exhaustive meta‐analysis of 22 RCTs from 1964 to 1986 evaluating the role of PMRT in women with one to three lymph node‐positive diseases. The meta‐analysis included all the studies included in this review. We excluded the remaining 19 studies due to their use of cobalt‐60 radiotherapy, having a target dose of more than 50Gy and not performing an axillary surgery to adequately stage the breast cancer. We based the permissible radiotherapy techniques in this review on those acceptable to the SUPREMO Trial to enable future comparisons to be made. We also excluded dose escalation (e.g. in the form of chest wall boost dose as there is no standard consensus for this approach). The EBCTCG 2014 of 1133 women who received some form of adjuvant systemic treatment (chemotherapy and endocrine) demonstrated that PMRT substantially reduces 10‐year local and regional recurrence (4.3% versus 21%, two‐tailed P < 0.00001) and improves 20‐year breast cancer‐specific mortality (41.5% versus 49.4%, RR 0.78, 95% CI 0.64 to 0.94, two‐tailed P = 0.01). Most of the women had systemic chemotherapy using the CMF regimen, systemic treatments involving ovarian irradiation were administered in two studies and systemic endocrine (tamoxifen) treatment was administered only in three studies. Hence, similar to our results, care should be taken while interpreting the magnitude of these results due to the difference in the effectiveness of the systemic treatment used in the included studies compared to that of the modern chemo‐endocrine agents.

Headon 2016 presented a meta‐analysis combining retrospective cohort studies and RCTs evaluating the effect of PMRT in women with one to three positive axillary lymph nodes. The local and regional recurrence analysis included 11 studies, and only 2 of these were RCTs. The remaining 9 were retrospective studies. The two RCTs included in the local and regional recurrence analysis were Overgaard 2007 and Ragaz 2005, with a total weightage of RCTs in the analysis being 59%. There was a statistically significant lower local and regional recurrence amongst PMRT women compared to no PMRT (RR 0.30, 95% CI 0.23 to 0.38). We excluded Ragaz 2005 from our review as they used cobalt‐60 radiotherapy, which is not considered an international standard of care moving forward The overall survival analysis involved only a single RCT (Ragaz 2005) with a weightage of 3.5% and showed a 3% improvement in overall survival amongst the PMRT group but did not reach statistical significance.

## Authors' conclusions

**Implications for practice**

Based on one study, this review showed a reduction in locoregional recurrence and improvement in overall survival with the use of postmastectomy radiotherapy (PMRT) amongst women diagnosed with breast cancer and found to have one to three positive axillary lymph nodes. There is a lack of published evidence to draw conclusions on the side effects and quality of life outcome measures after PMRT.

**Implications for research**

There is currently a paucity of evidence within the literature to evaluate the effectiveness of PMRT in women diagnosed with breast cancer and low‐volume axillary disease. Currently, there is a single ongoing multicentre international randomised study that is trying to address this question and could add further insight into the role of PMRT in women with low‐volume axillary disease. Despite this, it is likely that further studies in the future will still be needed to address the same question with updated current radiotherapy techniques to truly evaluate the benefit of treatment in view of the reduction in late adverse effects these newer techniques have to offer.

## History

Protocol first published: Issue 9, 2021

**Table:** 

**Date**	**Event**	**Description**
2 October 2021	Amended	Updated work affiliation.

## Appendices

### Appendix 1. CENTRAL search strategy

#1 MeSH descriptor: [Breast Neoplasms] explode all trees #2 breast near neoplasm* #3 breast near carcinoma* #4 breast near cancer* #5 breast near tumour* #6 breast near tumor* #7 #1 or #2 or #3 or #4 or #5 or #6 #8 MeSH descriptor: [Mastectomy] explode all trees #9 mastectom* #10 #8 or #9 #11 MeSH descriptor: [Radiotherapy] explode all trees #12 MeSH descriptor: [Radiotherapy, Adjuvant] explode all trees #13 radiotherap* #14 irradiat* #15 post‐mastectomy NEAR radiotherap* #16 postmastectomy NEAR radiotherap* #17 PMRT #18 post‐mastectomy NEAR radiat* #19 postmastectomy NEAR radiat* #20 radiation NEAR therap* #21 post‐operative NEAR radiotherap* #22 postoperative NEAR radiotherap* #23 post‐operative NEAR radiat* #24 postoperative NEAR radiat* #25 {OR #11‐#24} #26 #7 AND #10 AND #25 in Trials

### Appendix 2. Medline Ovid search strategy

**Table:**

#	**Searches**
1	randomized controlled trial.pt.
2	controlled clinical trial.pt.
3	randomized.ab.
4	placebo.ab.
5	Clinical Trials as Topic/
6	randomly.ab.
7	trial.ti.
8	(crossover or cross‐over).tw.
9	Pragmatic Clinical Trials as Topic/
10	pragmatic clinical trial.pt.
11	or/1‐10
12	exp Breast Neoplasms/
13	(breast adj6 cancer$).tw.
14	(breast adj6 neoplasm$).tw.
15	(breast adj6 carcinoma$).tw.
16	(breast adj6 tumo?r$).tw.
17	or/12‐16
18	exp Mastectomy/
19	mastectom*.tw.
20	or/18‐19
21	exp Radiotherapy/
22	exp Radiotherapy, Adjuvant/
23	radiotherap*.tw.
24	post‐mastectomy radiotherap*.tw.
25	post mastectomy radiotherap*.tw.
26	postmastectomy radiotherap*.tw.
27	PMRT.tw.
28	post‐mastectomy radiat*.tw.
29	post mastectomy radiat*.tw.
30	postmastectomy radiat*.tw.
31	radiation therap*.tw.
32	post‐operative radiotherap*.tw.
33	post operative radiotherap*.tw.
34	postoperative radiotherap*.tw.
35	post‐operative radiat*.tw.
36	post operative radiat*.tw.
37	postoperative radiat*.tw.
38	irradiat*.tw.
39	or/21‐38
40	11 and 17 and 20 and 39
41	animals/ not humans/
42	40 not 41
43	remove duplicates from 42

### Appendix 3. Embase Ovid search strategy

**Table:**

#	**Searches**
1	Randomized controlled trial/
2	Controlled clinical study/
3	Random$.ti,ab.
4	randomization/
5	intermethod comparison/
6	placebo.ti,ab.
7	(compare or compared or comparison).ti.
8	(open adj label).ti,ab.
9	((double or single or doubly or singly) adj (blind or blinded or blindly)).ti,ab.
10	double blind procedure/
11	parallel group$1.ti,ab.
12	(crossover or cross over).ti,ab.
13	((assign$ or match or matched or allocation) adj5 (alternate or group$1 or intervention$1 or patient$1 or subject$1 or participant$1)).ti,ab.
14	(assigned or allocated).ti,ab.
15	(controlled adj7 (study or design or trial)).ti,ab.
16	(volunteer or volunteers).ti,ab.
17	trial.ti.
18	or/1‐17
19	exp breast/
20	exp breast disease/
21	(19 or 20) and exp neoplasm/
22	exp breast tumor/
23	exp breast cancer/
24	exp breast carcinoma/
25	(breast$ adj5 (neoplas$ or cancer$ or carcin$ or tumo$ or metasta$ or malig$)).ti,ab.
26	or/21‐25
27	exp mastectomy/
28	mastectom*.tw.
29	or/27‐28
30	exp radiotherapy/
31	exp adjuvant radiotherapy/
32	radiotherap*.tw.
33	post‐mastectomy radiotherap*.tw.
34	post mastectomy radiotherap*.tw.
35	postmastectomy radiotherap*.tw.
36	PMRT.tw.
37	post‐mastectomy radiat*.tw.
38	post mastectomy radiat*.tw.
39	postmastectomy radiat*.tw.
40	radiation therap*.tw.
41	post‐operative radiotherap*.tw.
42	post operative radiotherap*.tw.
43	postoperative radiotherap*.tw.
44	post‐operative radiat*.tw.
45	post operative radiat*.tw.
46	postoperative radiat*.tw.
47	irradiat*.tw.
48	exp irradiation/
49	or/30‐48
50	18 and 26 and 29 and 49
51	limit 50 to (human and (conference abstracts or embase))
52	remove duplicates from 51

### Appendix 4. WHO ICTRP search strategy

#### Basic search

1. breast cancer AND post‐mastectomy AND radiotherapy OR breast cancer AND postmastectomy AND radiotherapy

2. breast cancer AND post‐mastectomy AND radiation OR breast cancer AND postmastectomy AND radiation

3. breast cancer AND post‐operative AND radiotherapy OR breast cancer AND postoperative AND radiotherapy

4. breast cancer AND post‐operative AND radiation OR breast cancer AND postoperative AND radiation

#### Advanced search

1. Condition: Breast cancer Intervention: post‐mastectomy AND radiotherapy OR postmastectomy AND radiotherapy Recruitment status: All

2. Condition: Breast cancer Intervention: post‐mastectomy AND radiation OR postmastectomy AND radiation Recruitment status: All

3. Condition: Breast cancer Intervention: post‐operative AND radiotherapy OR postoperative AND radiotherapy Recruitment status: All

4. Condition: Breast cancer Intervention: post‐operative AND radiation OR postoperative AND radiation Recruitment status: All

### Appendix 5. ClinicalTrials.gov search strategy

#### Basic search

1. Condition or disease: breast cancer Other terms: post‐mastectomy radiotherapy

2. Other terms: PMRT

#### Advanced search

1. Condition or disease: Breast cancer Intervention/treatment: post‐mastectomy radiotherapy OR postmastectomy radiotherapy

2. Condition or disease: Breast cancer Intervention/treatment: post‐mastectomy radiation OR postmastectomy radiation

3. Condition or disease: Breast cancer Intervention/treatment: post‐operative radiotherapy OR postoperative radiotherapy

4. Condition or disease: Breast cancer Intervention/treatment: post‐operative radiation OR postoperative radiation

## Data and analyses

**Comparison 1:** PMRT versus no PMRT in women with one to three positive lymph nodes

Outcome or subgroup title	No. of studies	No. of participants	Statistical method	Effect size
1.1 Locoregional recurrence (LRR)	1	552	Hazard Ratio (IV, Random, 95% CI)	0.20 [0.13, 0.33]
1.2 Overall survival (OS)	1	552	Hazard Ratio (IV, Random, 95% CI)	0.76 [0.60, 0.97]
1.3 Disease‐free survival (DFS)	1	173	Subdistribution Hazard Ratio (IV, Random, 95% CI)	0.63 [0.41, 0.96]

## Characteristics of studies

### Characteristics of included studies [ordered by study ID]

**Table:** Killander 2007

***Study characteristics***		
Methods	The South Sweden Breast Cancer Group (SSBCG II:1) randomly assigned postmenopausal women to postmastectomy radiotherapy (PMRT) or no radiotherapy (no PMRT) after surgery. The randomisation was stratified on the department, tumour size and the number of positive axillary nodes (N0, N1‐3 and N ≥ 4).	
Participants	Postmenopausal women (defined as at least 5 years of amenorrhoea) aged 71 years or below with stage 2 invasive breast cancer were recruited from all the 15 surgical departments in South Sweden. They underwent modified radical mastectomy with en‐bloc dissection of level 1 and 2 lymph nodes in the axilla prior to randomisation. Exclusion criteria: Nonradical surgery Other malignant diseases except for squamous cell cancer of the skin or cervical cancer in situ Bilateral breast cancer	
Interventions	Women were randomised into: Postoperative radiotherapy only (n = 239); Radiotherapy with tamoxifen (postmastectomy radiotherapy (**PMRT**)) (n = 234); and Tamoxifen‐only arms (**no PMRT)** (n = 251). The radiotherapy was administered using a ventral photon beam with the maximum targeted skin dose of 45 Gy administered in 20 fractions over a period of 5 weeks. The target volume consists of 4 subvolumes (i.e. the lymph nodes in the supra‐ and infraclavicular fossae, axilla, chest wall and the ipsilateral parasternal mammary lymph nodes). PMRT and no‐PMRT groups were offered tamoxifen as adjuvant treatment (30 mg/day for 1 year). The number of women with 1 to 3 positive lymph nodes that were randomised and included in this analysis: Radiotherapy with tamoxifen (**PMRT**) = 79 Tamoxifen only (**no PMRT**) = 94	
Outcomes	Locoregional recurrence (the occurrence of systemic disease and death were competing events) Time to systemic disease (the equivalent of disease‐free survival) Time to the first event of the distant disease or death from breast cancer (the occurrence of non‐breast cancer death was a competing event) Overall mortality was reported (instead of overall survival) No distinction was made between primary and secondary endpoints.	
Notes		
***Risk of bias***		
**Bias**	**Authors' judgement**	**Support for judgement**
Random sequence generation (selection bias)	Low risk	The randomization was stratiﬁed on department, tumour size and number of positive axillary lymph nodes with block size of 6 within strata.
Allocation concealment (selection bias)	Low risk	It was performed by calling the South Sweden Breast Cancer Group’s central secretariat, at which a closed envelope with a prerandomised allocation was selected. The identity of the participant, date, department and allocated treatment were documented by the secretariat.
Blinding of participants and personnel (performance bias) All outcomes	Unclear risk	A lack of blinding of participants and personnel was not considered to be a serious concern given the objective nature of the outcomes reported
Blinding of outcome assessment (detection bias) All outcomes	Unclear risk	A lack of blinding of outcome assessment was not considered to be a serious concern of bias given the objective nature of the outcomes reported
Incomplete outcome data (attrition bias) All outcomes	Low risk	Fifty‐six out of 724 participants randomised (7.7%) were lost to follow‐up. A clear definition of missing data and loss of follow‐up was depicted in the CONSORT diagram (figure 1 in trial publication).
Selective reporting (reporting bias)	Low risk	All relevant primary outcome measures (local and regional recurrence, disease‐free survival and overall survival) were considered and adequately defined. The long‐term outcome measures including side effects described in the paper have been collected retrospectively and have not been powered. Also, there is no representation of data specific to the N1‐N3 category of women for the long‐term outcome measures or side effects.
Other bias	Low risk	By mistake, the secretariat did not record the allocated treatment for the ﬁrst 47 participants but only date, their identity and institution. The adjuvant treatment with tamoxifen was given irrespective of the oestrogen receptor status.

**Table:** Killander 2009

***Study characteristics***		
Methods	The South Sweden Breast Cancer Group (SSBCG) randomly assigned premenopausal women to postmastectomy radiotherapy (PMRT) or no radiotherapy (no PMRT) after surgery. The randomisation was stratified on department, tumour size and the number of positive axillary nodes (N0, N1‐3 and N ≥ 4).	
Participants	Premenopausal women with stage 2 invasive breast cancer were recruited from all the 15 surgical departments in South Sweden. They underwent modified radical mastectomy with en‐bloc dissection of level 1 and 2 lymph nodes in the axilla prior to randomisation. Exclusion criteria Nonradical surgery Other malignant diseases except for squamous cell cancer of the skin or cervical cancer in situ Bilateral breast cancer	
Interventions	Women were randomised into: Postoperative radiotherapy only (n = 134); Radiotherapy and cyclophosphamide (postmastectomy radiotherapy (**PMRT**)) (n = 125); and Cyclophosphamide‐only arms (**no PMRT)** (n = 136). The radiotherapy was administered using a ventral photon beam with the maximum targeted skin dose of 45 Gy administered in 20 fractions over a period of 5 weeks. The target volume consists of four subvolumes (i.e. the lymph nodes in the supra‐ and infraclavicular fossae, axilla, chest wall and the ipsilateral parasternal mammary lymph nodes). PMRT and no‐PMRT groups were offered 12 cycles of 130 mg/m^2^ oral cyclophosphamide (chemotherapy) 1 to 14 days in 28‐day cycles. The number of women with 1 to 3 positive lymph nodes that were randomised. Radiotherapy with tamoxifen (**PMRT**) = 54 Tamoxifen only (**no PMRT**) = 50	
Outcomes	Locoregional recurrence (the occurrence of systemic disease and death were competing events) illustrated using a cumulative incidence curve Time to systemic disease (the equivalent of disease‐free survival) Time to the first event of the distant disease or death from breast cancer (the occurrence of non‐breast cancer death was a competing event) illustrated using a cumulative incidence curve Overall mortality reported (instead of overall survival) using cumulative mortality curves No distinction was made between primary and secondary endpoints.	
Notes		
***Risk of bias***		
**Bias**	**Authors' judgement**	**Support for judgement**
Random sequence generation (selection bias)	Low risk	Stratified randomisation based on department, tumour size and number of lymph nodes with block size of 6 with the strata
Allocation concealment (selection bias)	Low risk	Was performed by calling the South Sweden Breast Cancer Group’s central secretariat, at which a closed envelope with a prerandomised allocation was selected. The identity of the participant, date, department and allocated treatment were documented by the secretariat.
Blinding of participants and personnel (performance bias) All outcomes	Unclear risk	A lack of blinding of participants and personnel was not considered to be a serious concern given given the objective nature of the outcomes reported
Blinding of outcome assessment (detection bias) All outcomes	Unclear risk	A lack of blinding for outcome assessment was not considered to be a serious concern of bias given the objective nature of the outcomes reported
Incomplete outcome data (attrition bias) All outcomes	Low risk	Attrition was low (< 5%) in the randomised participants. In one institution, more than 80% of the randomised women’s records were destroyed. Since the missing records very well might differ systematically from those retained, all participants from this institution were excluded (5% of all randomised women), except in analyses of overall survival. Follow‐up of all the selected group of participants from the initial randomised group was complete (N1‐N3)
Selective reporting (reporting bias)	Low risk	All endpoints were analyzed according to the intention‐to‐treat principle. Survival and other time‐to‐event endpoints were determined from date of randomization and analyzed by survival analysis techniques. Times to locoregional recurrence and systemic disease are illustrated by means of cumulative incidence curves, considering the competing risks of other events.
Other bias	Low risk	One arm of the 1:1:1 randomisation involved giving oral cyclophospamide alone as adjuvant treatment. During interim review due to the high incidence of event, this arm of the study was terminated in June 1983.

**Table:** Overgaard 2007

***Study characteristics***		
Methods	The Danish Breast Cancer Cooperative Group (DBCG) enrolled a total number of 3083 women into their 82b (premenopausal and menopausal, n = 1708) and 82c (postmenopausal and below 70 years, n = 1375) randomised controlled trials (RCTs). All women underwent a total mastectomy and removal of level 1 and partly level 2 axillary lymph nodes. Participants were then randomised to postoperative radiotherapy (postmastectomy radiotherapy (PMRT)) or no radiotherapy (no PMRT).	
Participants	A subgroup of 552 women who were randomised and met the criteria of 8 or more axillary lymph nodes removed at the time of surgery and found to have 1 to 3 positive nodes was selected for this study. Inclusion criteria: High‐risk breast cancer is defined as women with breast cancer that was node‐positive or T3 or T4 tumour or skin or deep fascia invasion. No evidence of metastatic distant disease from physical examination, biochemical tests, chest radiography and bone scintigraphy or bone radiography No previous or concomitant other malignant diseases Exclusion criteria: Women with macroscopic residual tumour	
Interventions	After surgery randomised to: Postmastectomy radiotherapy (**PMRT**) = 276; and No postmastectomy radiotherapy (**no PMRT**) = 276. Radiotherapy was administered to most women using a linear accelerator with a target volume of 50 Gy in 25 fractions over 35 days or 48 Gy in 22 fractions over 38 days. The radiotherapy was administered to the chest wall and regional nodes (axilla, internal mammary and supra‐ and infra‐clavicular nodes). The systemic adjuvant therapy was offered based on the menopausal status. All the premenopausal and menopausal women received 8 to 9 cycles of cyclophosphamide, methotrexate and 5‐fluorouracil (CMF; 600, 40 and 600 mg/m^2^, respectively) intravenously every 4 weeks for 9 months, whereas for 48 weeks 30 mg oral tamoxifen was given daily for the postmenopausal women.	
Outcomes	Overall survival Locoregional recurrence (without simultaneous distant recurrence)	
Notes		
***Risk of bias***		
**Bias**	**Authors' judgement**	**Support for judgement**
Random sequence generation (selection bias)	High risk	Described in the Overgaard 1997 paper. Only a closed envelope in the recruiting department was used for randomisation, and no stratification was performed for randomisation.
Allocation concealment (selection bias)	Low risk	Described in the Overgaard 1997 paper. A closed envelope in the recruiting department was used for randomisation, and no stratification was performed for randomisation.
Blinding of participants and personnel (performance bias) All outcomes	Unclear risk	A lack of blinding of participants and personnel was not considered to be a serious concern given the objective nature of the outcomes reported
Blinding of outcome assessment (detection bias) All outcomes	Unclear risk	A lack of blinding of for outcome assessment was not considered to be a serious concern of bias given the objective nature of the outcomes reported
Incomplete outcome data (attrition bias) All outcomes	Low risk	ITT analysis was performed. A clear definition of the excluded women was described. There was no issue with attrition as this was a highly selected group with more than 8 lymph nodes removed from the initial randomised cohort.
Selective reporting (reporting bias)	Low risk	A subgroup of 1152 women who met the criteria of having 8 or more nodes removed (above the median value) and who were node positive.
Other bias	High risk	A potential source of selction bias due to the inclusion of participants considered to be advanced disease rather than early breast cancer. Only a small number of women were T3 disease, and no one was T4. Four per cent and 5% in the RT and no‐RT groups.

M: whether the cancer has metastasised n: number of women N: number of lymph nodes that have cancer PMRT: postmastectomy radiotherapy T: size of the tumour

### Characteristics of excluded studies [ordered by study ID]

**Table:**

Study	Reason for exclusion
Arriagada 1995	Ineligible radiotherapy intervention ‐ Gamma rays were used for administering radiotherapy to the axilla and supraclavicular nodal failure. X‐ray (electrons) was used only in the chest wall and internal mammary nodes.
Baum 1980	Ineligible surgical intervention ‐ No axillary surgery was performed.
Cahlon 2015	Ineligible study population ‐ There were no data specific to randomised women with 1 to 3 involved nodes. The study authors were unable to provide the individual data for the randomised women with 1 to 3 involved lymph nodes.
Friedl 1983	Ineligible radiotherapy intervention ‐ Does not comply with the radiotherapy inclusion criteria. Randomised women were given a total dose of 60 Gy or 6000 rad.
Gustavsson 1999	Ineligible study design ‐ A highly selected sample from the randomised cohort was selected for performing this analysis.
Houghton 1994	Ineligible surgical intervention ‐ No axillary surgery was performed as part of the breast cancer treatment and hence does not meet the inclusion criteria.
Højris 1999	Ineligible study population ‐ The entire radiotherapy versus no‐radiotherapy cohort has been analysed without data specific to women with 1 to 3 involved nodes.
Højris 2000	Ineligible study design ‐ The study includes only a selective group of participants who were initially randomised from a single centre.
Killander 2014	Ineligible study population ‐ The study reported on the cardiac and cerebrovascular adverse events for the entire cohort of women (i.e. N0, N1 and N2). There are no data on randomised women with one to three involved lymph nodes for any of the outcomes (overall mortality, breast cancer‐specific mortality and morbidity, mortality and morbidity from heart disease and cerebrovascular disease).
Morgan 2002	Ineligible surgical intervention ‐ Women included in this study did not undergo adequate axillary staging procedure. One node from the lower axilla, one node from the apex and one node from the second intercostal space as internal mammary node were removed at the time of mastectomy. If no nodes were positive, they were classified as Stage A, low axillary node positive as Stage B and, if either apical or internal mammary node, positive as Stage C.
Nielsen 2006	Ineligible study population ‐ There are no data available on women with 1 to 3 involved lymph nodes.
Osman 2014	Ineligible study design ‐ Detailed methodology of randomisation process not available. Not clear whether the study was randomised or just a controlled study with the comparison group. Multiple attempts were made to contact the study authors without any success.
Overgaard 2020	Ineligible study population ‐ No separate data on N1 disease were published. The data for the entire randomised cohort were published. An attempt was made to contact the study authors to request data for participants with N1 disease without any success.
Ragaz 2005	Ineligible radiotherapy intervention ‐ Radiotherapy was given using gamma rays.
Rutqvist 1990	Duplicate publication ‐ Earlier results before the full follow‐up was completed. Ineligible intervention ‐ Gamma rays were used for administering radiotherapy to the axilla and supraclavicular nodal failure. X‐ray (electrons) was used only in the chest wall and internal mammary nodes.
Rutqvist 1992	Ineligible radiotherapy intervention ‐ Gamma rays were used for administering radiotherapy to the axilla and supraclavicular nodal failure. X‐ray (electrons) was used only in the chest wall and internal mammary nodes.
Rutqvist 2006	Ineligible radiotherapy intervention ‐ Gamma rays were used for administering radiotherapy to the axilla and supraclavicular nodal failure. X‐ray (electrons) was used only in the chest wall and internal mammary nodes.
Schmoor 2000	Ineligible radiotherapy intervention ‐ Most of the women received postmastectomy radiotherapy (PMRT) using gamma rays with telecobalt. The corresponding study author was contacted to confirm the type of radiotherapy being used in the study population.
Seegenschmiedt 2000	Duplicate publication ‐ There were no separate outcome data for randomised women with N1‐3 disease. Duplicate publication in German.
Shi 2011	Ineligible study design ‐ Unable to verify randomisation and study methodology. Multiple attempts were made to contact the study authors without any success.
Smith 2001	Study terminated due to failure to accrue.

### Characteristics of ongoing studies [ordered by study ID]

**Table:** Velikova 2018

Study name	SUPREMO Trial
Methods	Open‐label, parallel‐group, randomised, controlled trial
Participants	Women aged 18 years or older who had undergone mastectomy for unilateral breast cancer. Women were eligible if they had intermediate‐risk breast cancer (defined as pT1–2N1; pT3N0 or pT2N0 if also grade III or with lymphovascular invasion on histology). Participants needed to be fit for surgery, radiotherapy or adjuvant systemic therapy. Exclusion criteria included previous or concurrent malignancy (apart from nonmelanomatous skin cancer and carcinoma in situ of the cervix), ductal carcinoma in situ, bilateral breast cancer, pregnancy at the time of radiotherapy treatment and male sex.
Interventions	Chest‐wall radiotherapy or no chest‐wall radiotherapy
Outcomes	In this publication, only the quality of life secondary outcome measures were considered for the entire cohort of randomised women. The primary outcome measure was 10‐year overall survival. Quality of life is a secondary endpoint alongside chest wall recurrence, regional recurrence, disease‐free survival, acute and late morbidity, cost‐effectiveness, metastasis‐free survival and cause of death.
Starting date	4 August 2006 to 29 April 2013 ‐ Currently undergoing data collection and follow‐up
Contact information	Prof Galina Velikova, Leeds Institute of Cancer andPathology, University of Leeds, Leeds Cancer Centre, St James’s University Hospital, Leeds LS9 7TF, UK g.velikova@leeds.ac.uk Author was contacted to gather data specific to N1‐3 disease patients as the data published in this paper included both N1 and N0 patients.
Notes	In 2011, the eligibility criteria for the SUPREMO Trial was widened following a protocol amendment (version 29; 30 August 2010) approved by the ethics committee to include the use of neoadjuvant chemotherapy according to contemporary guidelines. As per meta‐analysis exclusion criteria, women having neoadjuvant chemotherapy should have to be excluded from the analysis.

## Differences between protocol and review

We adhered to the methods outlined in the published protocol and no changes were made during the production of this review.
